# Not in their hands only: hospital hygiene, evidence and collective moral responsibility

**DOI:** 10.1007/s11019-022-10120-0

**Published:** 2022-11-05

**Authors:** Saana Jukola, Mariacarla Gadebusch Bondio

**Affiliations:** 1 Institute for Medical Humanities, University of Bonn, Bonn, Germany; 2 Department of Philosophy I, Ruhr-University Bochum, Bochum, Germany

**Keywords:** Hospital acquired infections, Evidence, Moral responsibility, Collective responsibility, Medical ethics

## Abstract

Hospital acquired infections (HAIs) are a major threat to patient safety. This paper addresses the following question: given what is known about the causes of and possible interventions on HAIs, to whom or what should the moral responsibility for preventing these infections be attributed? First, we show how generating robust evidence on the effectiveness of preventive hygiene measures is a complex endeavour and review the existing evidence on the causes of HAIs. Second, we demonstrate that the existing literature on the ethical aspects of infection control has focused on responsibility at the individual-level. Thirdly, we argue that these accounts do not accommodate systemic factors relevant for HAI prevention. We show that the notion of collective responsibility is useful for making understandable how systemic factors, such as employment conditions in hospitals, are both causally and ethically relevant in infection control.

## Introduction

Hospital acquired infections (HAIs) are a major threat to patient safety. There are more than 2,000,000 new cases of HAI every year in the European Union and the European Economic Area (Cassini et al. 2016, p. 2) and the estimated annual costs of HAIs in the US are $9.8 billion (Zimlichman et al. 2013, p. 2039). Despite the considerable negative consequences of HAIs, the field of hospital infection control has attracted little research funding and attention among medical scientists until relatively recently (Siani and Maillard 2015, p. 1). Even though there have been specialized infection control nurses since the 1950s, infection prevention and control did not become an independent medical specialty with guidelines, policies and hired staff before the mid-1980s (Dancer 2016, p. 148). In the last couple of decades, however, literature on the field has increased. At the same time, researchers have gained a better understanding of the factors that contribute to infection rates in the hospital setting.

This paper addresses the following question: Given what is known about the causes of and possible interventions on HAIs, to whom or what should the moral responsibility for preventing these infections be attributed? The existing literature on the ethical aspects of infection control has focused on responsibility at the individual-level. Our main claim is that these accounts have to be complemented with analyses that address what we call the systemic determinants of HAIs. We argue that the notion of collective responsibility is useful for making understandable how systemic factors, such as employment conditions in hospitals, are both causally and ethically relevant in infection control. In order to make this claim, we show how the current evidence implies that individual-level prevention measures, such as hand hygiene, are insufficient for preventing HAIs. This deems previous medical ethical accounts of moral responsibility for HAI prevention insufficient. By drawing on discussion on collective responsibility (e.g., French 1982; List and Pettit 2011; Isaacs 2014), we then argue that hospitals as organizations should be held morally responsible for decisions that will increase HAI rates.

Before proceeding, a caveat is in order. When discussing factors relevant to infection control in hospital settings, we focus on actors and mechanisms inside hospital organizations. In other words, we will exclude, for example, broader societal factors such as economic growth or recession, cultural norms and health policy. We are aware that practices within hospitals often are dependent on such external influences: Hospitals are not fully autonomous organizations (cf. Tuomela and Mäkelä 2020, p. 376). They can be described as porous entities, embedded in particular economic and political contexts. For instance, public hospitals receive funding from state budgets and sudden epidemics force hospital organizations to adjust their processes. Moreover, given the differences in healthcare systems, external factors relevant to how hospitals function vary. Consequently, naming all responsible actors and determining their obligations would require a detailed analysis of each particular case. Instead of providing such an analysis here, our aim is to point towards a gap that exists in the current literature and show how the concept of collective responsibility can be used for addressing ethical issues related to infection control.

The structure of the paper is as follows. We begin by providing an outline of the research on hospital infection control. We first discuss how hand hygiene has been established as a central intervention. After this, we show how new research methods have led to the recognition of the relevance of environmental cleaning and disinfection in infection control. Finally, we discuss the evidence demonstrating how systemic factors, such as employment conditions, are associated with HAI rates. As this review shows, it is now known that promoting hand hygiene is an insufficient intervention on HAIs. Having outlined the existing evidence concerning means of HAI control, we address the question of moral responsibility for HAI prevention. First, we review existing literature on the topic. Here we show that the problem has been addressed in individual-centered terms. We argue that in the light of existing empirical evidence on HAIs, these accounts do not capture some central elements of infection control. We then move to discussing the concept of collective responsibility and show how it can help to address the lacunae left open by the individual-centered accounts. We finish the paper with some prescriptive notes concerning what individual and collective responsibility for infection control mean in practice.

## The challenge of hospital infection control: what is the evidence?

The importance of infection control in promoting health has been recognized for centuries. Two early pioneers of this field were Ignaz Semmelweis (1818–1865) and Florence Nightingale (1820–1910), who conducted their work in the nineteenth century. Many medical articles addressing hospital infection control discuss Semmelweis’s work, especially his realization that handwashing helped to reduce the rate of childbed fever and post-delivery mortality in a Viennese hospital where medical students were both handling cadavers and helping in deliveries. Nightingale is a less frequently discussed but nowadays rediscovered pioneer of hospital hygiene. She was one of the first to acknowledge the impact that a clean environment has on patient health (Gilbert 2020; Miracle 2008). The practices she implemented considerably reduced death rates in her hospital during the Crimean war in the 1850s (Fee and Garofalo 2010, p. 1591).

Despite this long history of hygiene control, HAIs result in considerable morbidity and mortality even today. Studies have shown at least half of these cases to be preventable (Zimlichman et al. 2013, p. 2039). Regardless of this, hygiene practices in hospitals have been based more on “common sense” than systematically collected scientific evidence (Dancer 2016, p. 148). While the danger of infections caused by pathogens has been acknowledged, assessing the means of preventing their spread has not been a hot topic for researchers and funders (Siani and Maillard 2015, p. 1). Consequently, the accumulation of evidence for the effectiveness of different measures has been piecemeal (Dancer 2016, p. 148).

This slowness in the accumulation of knowledge on how to best prevent HAIs is partially due to the multifactorial nature of infections and the related methodological challenges in research (Backman et al. 2008, p. 333; Dancer and Kramer 2019, p. 6, Elkomy et al. 2019, pp. 195–196). The evidence for the effectiveness of infection control measures has seldom been acquired via studies that rank high on the evidence-based medicine hierarchy of evidence, i.e., randomized controlled trials (Donskey 2013, p. 519).^1^ However, some scholars have also claimed that the lack of studies reflects uncritical and unreflective attitudes concerning hygiene: The connection between dirt and the presence of pathogens has not been adequately understood by those in charge of hospital management (Dancer 2008, p. 101). Nonetheless, during the last couple of decades, research in this area has expanded. In particular, the emergence of methicillin-resistant staphylococcus aureus (MRSA) has increased scientific and public interest in improving infection prevention in healthcare facilities (Dancer 2014, p. 667; Siani and Maillard 2015, p. 1).

In the next subsections, we show how the understanding concerning factors relevant to preventing infections in the hospital context has increased. Until recently, maintaining good hand hygiene was seen as the central means of preventing infections. Now the relevance of environmental cleaning and disinfection is also recognized. Moreover, growing body of evidence shows that HAI rates are associated with systemic factors, such as employment policies. In other words, the incidence of HAIs has been shown to be multifactorial.

### Hand hygiene and infection control

Until recently, the main route of pathogens from one patient to another was taken to be via the hands of healthcare workers (Beggs et al., 2006, p. 621; Dancer 2016, p. 148). According to Beggs et al. (2006, p. 621), this “has lead [sic] to the widespread opinion that HAI rates can be greatly reduced by increased hand hygiene compliance alone”. For example, the World Health Organization (WHO) states, “[t]here is clear evidence that hand hygiene multimodal improvement strategies are effective in improving practices and preventing microbial transmission and infections” (WHO 2017a, p. 7). Another WHO document notes, “[h]and hygiene performance according to [the WHO guideline recommendations] is the most important measure among standard precautions [for preventing the spread of micro-organisms]” (WHO 2017b, p. 1).

Reflecting this emphasis in the medical literature and guidelines, social scientific contributions related to infection control practices have focused on hand washing and disinfection (e.g., Mortel 2012; Gaube et al. 2018; Wichsova and Horakova 2018; Damilare 2020). Many of these studies have addressed the low rate of compliance with hand hygiene guidelines and tried to find means to improve it. In a review of 96 studies on compliance with guidelines, Erasmus et al. (2010) found the overall compliance rate [i.e., “the sum of all events in which hand hygiene was performed divided by the sum of all possible hand hygiene events” (p. 285)] to be 40%—and even lower among physicians.^2^ Other researchers even report hostile attitudes towards being reminded about hygiene policies (Jumaa 2005, p. 4; Gilbert and Kerridge 2019, p. 2 and 8). In order to improve HAI prevention, these studies suggest measures to influence the behaviour and attitudes of individuals. For example, Gilbert and Kerridge (2019, p. 7) recommend that clinicians should be better informed about the adverse effects of disobeying the hygiene rules. Similarly, Gaube et al. (2018) name hand hygiene as an effective method for preventing HAIs and propose that hygiene practices could be improved with the help of an “emoticon-based feedback system”, displaying a smiley after alcohol-based hand-rub has been used. In these contributions, HAI prevention is conceptualized as practice in which individuals, their attitudes and knowledge about the possible effects of their actions play a central role. Raising individuals’ awareness is taken to be essential for improving infection control practices and, thus, preventing HAIs.

It is worth noting that despite the abovementioned WHO’s position that the evidence for the effectiveness of hand hygiene in preventing HAIs is clear, there are voices questioning the quality of the available studies. For example, Bolton and McGullogh (2018) criticize WHO’s hand hygiene campaigns for being based on studies that use historical control data instead of contemporaneous control. Similarly, Backman et al. (2008, p. 244), who reviewed studies on hand hygiene published between 1996 and 2006, lamented that most of the studies were either before-and-after studies or ecological studies, and some contained methodological flaws. The problem of confounding in studies on hand washing and disinfecting has been noted by Dancer (2016, p. 148), who questions the claim that hand hygiene measures prevented the spread of MRSA in the UK when the infections were spreading in other countries. According to her, other measures, such as improved environmental cleaning and surveillance programmes, were introduced at the same time. Therefore, it is not clear what the real effect of hand hygiene was (Dancer 2016, p. 148). Despite these methodological and theoretical challenges in conducting studies on the effectiveness of hand hygiene, the importance of proper hand washing and disinfection has been hardly questioned. This is not the case with environmental cleaning and disinfection. The role of these measures in the fight against HAIs has been a much-debated issue.

### Environmental cleaning and infection control

Since the late 1990s, researchers interested in HAI prevention have broadened their perspective beyond hand hygiene, and more factors have now been associated with transmission of pathogens. As mentioned above, Florence Nightingale already recognized the importance of clean environment in the nineteenth century. However, since then the relevance of environmental cleaning (removal of dirt and soil) and surface disinfection in infection control was overlooked for a long period (Litwin et al. 2017, p. 635; Dancer and Kramer 2019, p. 5). Until recently it was believed that hospital environments were not a source of pathogens leading to HAIs (Siani and Maillard 2015, p. 1–2). There was also little research conducted in this area.

According to the literature, the reasons for the lack of research on environmental cleaning and disinfection as a means of preventing HAIs were two-fold. On the one hand, cleaning had not been considered to be an activity worthy of scientific study (Dancer 1999, p. 93). For example, according to Dancer (2008, p. 101), hospital environments have been assumed to be clean and, therefore, their possible influence on infection outbreaks was neglected. A second reason for the lack of research is that studying of the impact of environmental cleaning is methodologically challenging. Given that cleaning efforts have been difficult to standardize and their effectiveness difficult to measure, comparing individual studies has been demanding (Dancer and Kramer 2019, p. 6; Elkomy et al. 2019, p. 195). Case reports of outbreaks have been a typical form of evidence in this area. As the generalizability of these types of studies is questioned, their relevance for arguing for the importance of cleaning has often been challenged (Dancer 2014, p. 667).^3^ Moreover, some of the problems of acquiring evidence on the effects of cleaning were similar to those encountered in the studies on hand hygiene. Even quasi-experimental studies were conducted without adequate controls or follow-ups and thus confounding was difficult to rule out (Donskey 2013, p. 516). For example, studies on new cleaning products have often been carried out simultaneously with the introduction of other infection control measures (Donskey 2013, p. 514; Dancer 2016, p. 149). Determining which of the measures were responsible for the observed outcome has thus been problematic.

The dismissive attitudes towards the importance of cleaning started to change in the early 2000s when studies on the role of environmental contamination in pathogen transmission began to emerge (e.g., Weber et al. 2010, pp .25–26; Dancer 2008, pp. 379–380). A major factor in this development were the new research methods that enabled collecting data about the role that environmental contamination plays in HAI rates. In particular, two methodological advancements were significant in establishing that pathogens are transferred not only via patient-healthcare worker-patient chains but also via contaminated surfaces (Dancer 2016, p. 149). The first of these were the studies showing how dormant pathogens could cause several cases of infection in one patient room even months apart: Patients who had been accommodated in rooms where a previous patient had had an infection were more likely to contract the same infection (e.g., Mitchel et al. 2015). The development of Next Generation Sequencing techniques helped to establish that without adequate cleaning, pathogens may remain alive in dirt and cause an outbreak even long after the original patient with the infection has been released. For example, it is now known that MRSA mixed in hospital dust can stay dormant and cause new infections even 1 year after it has been introduced to an environment (Dancer 2008, p. 104).

The second methodological development significant for establishing the importance of cleaning enabled better measurement of cleaning success. Previously it had been usual to assess cleanliness visually, which deemed the measurements subjective and inaccurate (Elkomy et al. 2019, p. 196). Visually clean does not mean that invisible dirt and pathogens have been successfully removed (Siani and Maillard 2015, p. 4; Dancer 2014, p. 680). However, chemical (e.g., ATP bioluminescence) and microbiological (e.g., overall aerobic colony and specific pathogen colony count) techniques now make it possible to assess cleaning performance more objectively (Dancer 2014, p. 680; Elkomy et al. 2019, p. 196; Hall and Mitchell 2020, pp. 231–232).

Thanks to the growing body of evidence, cleaning and decontamination are now widely recognized as important preventive measures against HAIs (Dancer and Kramer 2019, p. e1). It is accepted that in order to prevent the spread of pathogens, it is not sufficient that individuals maintain good hand hygiene (e.g., by washing and disinfecting their hands between patient contacts) because transfer can happen via contaminated surfaces. As Dancer and Kramer (2019, p. 2) note, “even exemplary hand hygiene will not protect patients from acquiring a specific pathogen if their room was previously occupied by a patient with the same pathogen [and the room was not adequately cleaned]”. Improving the cleaning of patient rooms and other areas of hospitals is now known to help to block the routes of pathogen transmissions (Donskey 2013, p. s12). Florence Nightingale’s message, forgotten for decades, is at last being taken seriously again. Cleaning is important not only for aesthetic reasons but for improving patients’ health.

### Systemic factors and infection control

But what factors have an impact on the effectiveness of cleaning and surface disinfection? There is considerable evidence suggesting that the success of cleaning activities depends on what we here call systemic factors. For example, bed turnover rates, the availability of storage space and isolation rooms, and the time pressure the staff faces have all been associated with the quality of cleaning (Dancer 2014, p. 681; Harbarth et al. 1999, p. 601; Beggs et al. 2006, p. 621; Clements et al. 2008, p. 427). If staff members do not have enough time to clear away objects that hinder removing visible and invisible dirt, the risk of creating an environment conducive to pathogens increases (Dancer 2008, p. 4). Moreover, built-environmental features of a hospital impact cleaning success. Some materials that can be used as surfaces have antimicrobial properties and are thus easier to keep clean than others (Dalton et al. 2020, p. 10). Increased rate of interaction between staff and different patients (e.g., decreased cohorting^4^) is also linked to higher rates of pathogen transmission (Nijssen et al. 2003, pp. 2785–6). Further, many experts on hospital infection control recognize that some of the recent developments in healthcare systems have had a negative effect on hygiene control measures. In many countries, healthcare systems have been restructured to increase efficiency and cost-efficiency, which has had an impact on the conditions of adequate cleaning. For example, in the UK, increased patient admission rates and bed reductions have led to overcrowding and understaffing in hospitals (Clements et al. 2008). When patients are moved from one ward to another, the risk of contamination increases if rooms and surfaces cannot be adequately cleaned between patients. Yet cleaners often already have a heavy workload, they receive little training, and are poorly paid (Dancer and Kramer 2019, p. 2). As Dancer (2014, p. 681) has noted, “in an era of cost cutting, those with cleaning responsibilities cannot hope to decontaminate all high-risk surfaces as often as required when a hospital is full to capacity and patients with attendant microorganisms are transferred between wards (and hospitals) day and night”. The lack of time is a barrier to halting the spread of pathogens.

The increase in HAI cases is also linked to staffing practices. As Nijssen and colleagues (2003, p. 2786) note, “understaffing will affect almost all relevant variables of [pathogen] transmission dynamics”. Moreover, the outsourcing of environmental services has been shown to be related to higher infection rates. For instance, a study based on cross sectional data conducted by Litwin and colleagues (2017) showed that the outsourcing of environmental workers was associated with an increase in reported Clostridium difficile rates in California’s general acute care hospitals. Similarly, Elkomy and colleagues’ (2019) analysis of data from 130 English National Health Services trusts revealed that outsourced cleaning services were associated with lower cleaning standards and higher rates of MRSA infections.

Qualitative research provides evidence about the mechanisms that may be responsible for the association between outsourcing and increased HAI rates. For instance, Dan Zuberi’s (2013) ethnographic work in British Columbia describes the challenges that outsourced cleaning staff face. Overworked cleaners have few of the training and education opportunities needed for performing their tasks and often lack supplies required for doing the job properly (Zuberi 2013, ch. 3 and 4, see also Dancer and Kramer 2019). Zuberi further argues that outsourcing cleaning staff compromises the teamwork necessary for infection control: When cleaners report to the managers of their own company instead of hospital employees, the flow of information is disrupted—sometimes with risky consequences. For instance, the cleaners may not be informed that a particular patient has contracted a dangerous pathogen and that their room should not be entered without special protective gear (Zuberi 2013, p. 70). Given their precarious working situation and fear for losing their jobs, individuals interviewed by Zuberi (2013, ch. 6) reported being hesitant to report problems to their superiors. In other words, with the outsourcing of cleaning staff, an important line of communication and mutual control breaks down. These factors have a negative impact on how well pathogens can be removed from the hospital environment.

Unlike in the case of complying with hand hygiene guidelines, individual hospital employees, whether they are cleaners, nurses or doctors, have little control over bed occupation rates, how storage room is organized in hospital wards and what surface materials are used. The same applies to staff hours and employment relations. As we have shown above, evidence shows that these systemic factors have an impact on HAI rates and that improving individual-level determinants of HAIs, e.g., hand hygiene, alone is not sufficient for preventing infections. The ethical implications of this multifactorial nature of HAIs are discussed in the next section. In particular, we argue that the notion of individual responsibility cannot accommodate all determinants of HAIs. We suggest that it is therefore appropriate to apply the concept of collective responsibility to HAI prevention.

## Moral responsibility for infection control in hospitals

Failing to protect patients from harm that could be prevented is an ethical issue. Given the considerable harm that HAIs cause, hospital infection control has clear ethical dimensions (Gilbert and Kerridge 2019, p. 2). This raises the following question: To whom or what should the moral responsibility for preventing HAIs be attributed?

Responsibility is typically understood to include both causal and normative dimensions (e.g., Müller et al. 2021, p. 3; Miller 2020, p. 38). When attributing causal responsibility to an agent, we simply state that this agent contributed to an event taking place. For example, a doctor who does not disinfect their hands before a patient contact may be causally responsible for infecting the patient with a pathogen. Attributing moral responsibility, in turn, concerns assigning blame or praise for an act. Think about a doctor who infects a patient because they have not adequately disinfected their hands before conducting an emergency tracheotomy during a transatlantic flight, knowing that the first priority is to save lives. In this case the doctor may be causally responsible for the infection without being morally responsible for it (see, e.g., Miller 2020, p. 38).

When can an agent then be held morally responsible for an act? For instance, under what conditions should the doctor who has not washed and disinfected their hands be assigned moral responsibility for infecting the patient? There is a debate about whether it is possible to name a set of necessary and sufficient conditions for moral responsibility or obligation and if so, what those conditions are (Müller et al. 2021, p. 4; Brown and Savulescu 2019, p. 637).^5^ However, it is generally acknowledged that an agent cannot bear moral responsibility if they do not have control over their actions or lack an understanding of the potential consequences of those actions (List and Pettit 2011, p. 155; Brown and Savulescu 2019, p. 637; Müller et al. 2021, p. 3).^6^ In other words, the condition of control and some epistemic conditions must be satisfied before moral responsibility can be assigned (Müller et al. 2021, p. 3): an agent cannot be held morally responsible if they could not have acted differently—*ought* famously implies *can* (e.g., Collins 2013, p. 232). Moreover, accumulation of knowledge concerning causal responsibilities can change as to whom—or what—moral responsibility should be assigned.

Another aspect of moral responsibility is that it has backward-looking and forward-looking dimensions. The former applies to the assessment of an agent’s past actions, for example in connection to questions of guilt for past wrongs (Isaacs 2014, p. 43). The latter, in turn, refers to future actions and is sometimes discussed as collective obligation (Isaacs 2014, p. 44). As Müller et al. (2021, p. 4) note, accounts of forward-looking responsibility often concern questions related to the prevention of certain events. Consequently, they can be applied to analyzing ethical aspects of hospital infection control.

In the next subsections, we discuss moral responsibility for infection control. First, we show that the existing ethical literature approaches the topic from an individualistic perspective and assigns responsibility for preventing infections to individual healthcare workers. They are expected to follow guidelines, report if they observe any hygiene violations, and inform employers about obstacles (e.g., the lack of necessary equipment) for following the guidelines. We will argue that given that the current evidence about the multifactorial nature of HAIs, these individualistic approaches turn out to be insufficient. They cannot accommodate some of the central factors relevant for successful infection control and thus lead to what List and Pettit (2011, p. 165) have called a “deficit of responsibility”. We suggest that the notion of collective responsibility helps to account also for the systemic factors of infection control as morally relevant. By applying an account according to which moral responsibility can be assigned to organization satisfying certain criteria (e.g., French 1982; List and Pettit 2011; Isaacs 2014), we argue that hospitals bear moral responsibility for infection control.

### Individual moral responsibility for hospital infection control

Some of the ethical aspects of hospital infection control and HAI prevention have already been discussed in the previous literature. For example, Herwaldt (1996) addresses the choices that infection control personnel have to make in their day-to-day work and outlines how the principles of medical ethics and public health ethics apply to hospital epidemiology. While the problem of responsibility is not explicitly discussed by Herwaldt (1996), it is addressed, for example, by Gilbert et al. (2009), Silva and Ludwig (2006), Millar (2009), Wichsová and Horáková (2018) and Gadebusch Bondio (2022).^7^ Gilbert and colleagues (2009, p. 697) emphasize individuals’ responsibility to comply with the hygiene guidelines and suggest sanctions for those who fail to follow the rules. Similarly, Silva and Ludwig (2006) argue that nurses have a moral responsibility to protect both patients and themselves, which implies that they have to follow hygiene guidelines. Moreover, nurses are responsible for monitoring practices at their workplace and speaking up if they observe obstacles to achieving hygiene goals (Silva and Ludwig 2006). Responsibility for compliance with hygiene guidelines, combined with practiced vigilance and systematic reporting of omissions, is discussed in the specific context of the ethical dimensions of hygiene in the perioperative setting by Gadebusch Bondio (2022).

These authors delineate individuals’ responsibilities for following the hygiene guidelines on the basis of the four principles of medical ethics.^8^ Although the extent of responsibility varies depending on the function, position, and expertise of each individual, responsibility to self, colleagues, and patients is required of everyone. In dealing with patients, the principles of beneficence and non-harm are paramount for physicians and nurses. The duty to protect oneself and others from infections is also based on these principles of biomedical ethics. This, in turn, means that carelessness, negligence, and trivialization of HAI risks constitute conduct that is contrary to professional ethics, in that one endangers the safety and thus the well-being of others or tharms their health (Wichsová and Horáková 2018, p. 191; Gadebusch Bondio 2022).

As described in the previous section, due to improved methods and increased research interest in hospital infection control, the body of evidence concerning the causes of HAIs has grown. It is now known that hand washing and other individual-centered prevention measures are insufficient for preventing the spread of pathogens. This is because even if individuals did wash and disinfect their hands and otherwise follow the hygiene guidelines, pathogens remaining on dirty surfaces may infect patients. Moreover, studies have shown how systemic factors, such as employment conditions and bed occupation rates, influence the cleanliness of hospital environments—and consequently HAI rates. As Litwin and colleagues write in their study on Clostridium difficile rates in California’s general acute care hospitals (2017, p. 611), “the employment arrangements under which [cleaners] perform their tasks and that govern the manner in which they are managed, trained, compensated, and treated are likely to contribute to their preparedness and *ability* to [influence the spread of pathogens] [emphasis added]”. Especially overworked, underequipped and undertrained outsourced cleaners often do not have the resources to do their job properly. In overcrowded hospitals, the increased movement of patients between wards and decreased cohorting lead to more contacts between different nursing staff members and different patients, thus raising the number of possible transmissions (Nijssen et al. 2003, pp. 2785–2786). The ability of individuals to fulfil their duties while maintaining the standards required to prevent the spread of pathogens can be thus be hindered by their working conditions. What this implies is that individual hospital workers, even if they are aware of the importance of following the hygiene guidelines, often operate under conditions where they do not have adequate control over actions necessary for preventing infections. In other words, one of the conditions for assigning moral responsibility to them is not satisfied.

The fact that individual hospital workers cannot always follow the guidelines is acknowledged by some of the abovementioned authors addressing the ethical aspects of infection control. For example, when Wichsová and Horáková (2018, p. 194) name organization culture as a relevant factor in HAI prevention, they focus on how “the overall atmosphere should promote adherence to the rules”. Gilbert et al. (2009, p. 697) explicitly note that evidence shows HAI rates depend on systemic factors. However, they worry that acknowledging the role that system failures play in the spread of infections can direct attention away from individual healthcare workers’ responsibility for following hygiene guidelines (Gilbert et al. 2009, p. 698). Even though these authors acknowledge the relevance of organisational or systemic factors and the complex role of reciprocal responsibility, they assign the moral responsibility for infection prevention to individuals and the question of the structural conditions for compliance with hygiene guidelines is addressed only sporadically. This tendency to emphasize individual responsibility overlooks how individuals’ ability to satisfy the condition of control required for moral responsibility is undermined by their working conditions. Consequently, these individualistic accounts fail to capture or address some of the determinants of HAIs.^9^ For example, outsourcing cleaning staff is a decision that has an impact on infection rates, yet it is a decision that is seldom made by an individual. This points towards the need to broaden the focus of accounts assessing the ethical aspects of infection control. We next argue that the concept of collective responsibility is helpful in incorporating the systemic and organisational factors into ethical considerations.

### Collective responsibility for hospital infection control

The notion of collective responsibility denotes assigning causal responsibility and moral blameworthiness to collectives, for instance companies, universities, and other structured groups of people, distinctively from their individual members (Smiley 2017; Miller 2020, p. 39). In the context of hospital hygiene, our concern is to show that responsibility is to be considered also on a collective and not only on an individual-level. As Hormio (2017, p. 82) has noted, stating that collectives can have obligations that are independent of the individuals that belong to those collectives is uncontroversial. For instance, a corporation has obligations towards their shareholders no matter who the members of its board of directors are, and universities have the obligation to provide education to their students regardless of who works at the universities (Hormio 2017, p. 82). However, whether collectives also have responsibilities in a moral sense is more contested.

There are several accounts according to which collective agents, such as corporations, can also be included in the analyses of moral responsibility. Some of these have been applied in the context of healthcare, where groups such as professional associations and teams are responsible for actions in a way that cannot be fully captured by individual-centered accounts of responsibility. For example, the concept of individual responsibility has trouble accommodating cases where complex medical technologies are used by teams of professionals (Müller et al 2021, p. 3). The actions of such teams may result in harm, say the death of a patient, even if no individual team member could be held morally responsible for them (see, e.g., List and Pettit 2011, pp. 165–167). The concept of collective moral responsibility allows us to analyze the ethical aspects of such cases. It has to be noted that subcollectives can exist within bigger collectives. In this sense, the collective responsibilities multiply, according to the groups and subgroups within structures that make decisions for action.^10^

The question of what it means for a collective to be morally responsible has raised a lot of debate (Smiley 2017). According to some authors, the responsibility of a group is always an aggregate of the responsibilities of the members of that group, in other words *shared responsibility* (French 1982, p. 70; Smiley 2017; Müller et al. 2021, p. 4).^11^ In this framing of group responsibility, responsibility does not exist independently of discrete individuals. For instance, moral responsibility of a company would simply refer to the responsibilities of employees of the company. This position is connected to the doubt about whether collectives in general can have intentions and whether they are capable of acting, i.e., satisfying conditions of being held responsible (see Smiley 2017 for an overview of the debate). A contrary position is that in some cases the moral responsibility of the group cannot be reduced to the individual-level: The collective (e.g., a corporate body, a university etc.) can be held blameworthy for an act in a way that cannot be reduced to blameworthiness of individuals. For example, according to French (1982), List and Pettit (2011) and Isaacs (2014), moral responsibility can be assigned to groups independently of their individual members, especially to organizations that satisfy certain criteria, such as having a decision structure, which helps to determine how the organization acts to achieve shared goals.^12^ An organizational power structure and decision recognition rules provide guidelines for collective decision-making (French 1982, p. 72; List and Pettit 2011, ch. 7.2.; Isaacs 2014, p. 42). The notion of *collective responsibility* is used when referring to these cases.

According to French (1982, pp. 72–75), hospitals are paradigmatic examples of agents to which collective responsibility can be assigned.^13^ They are structured organizations, and have assigned positions with chains of command. These are the features needed for assigning corporate decisions and policies that direct future actions. Particularly in the case of groups that make decisions with far-reaching consequences (the board of directors of a hospital, health policy committees), collective responsibility is clearly given. Moreover, the identities of hospitals are not reducible to or consist of the identities of the individuals working at those hospitals. The Fair Hope Hospital is still the Fair Hope Hospital, even though some employees will resign or retire and new ones will be hired. Hospitals are thus morally responsible agents making strategic choices that can be praised or condemned and not reduced to the actions of any individuals (French 1982, p. 72; Müller et al. 2021, p. 5). Consequently, statements such as “Fair Hope Hospital is to blame for understaffing the nursing surveillance stations in the Intensive Care Ward” (French 1982, p. 72) can be used for assigning moral responsibility for a harm that has happened, even though no individual working at the hospital could be held responsible for it.

Collective and individual responsibility operate at different levels, and adding the notion of collective responsibility to ethical analysis is meant to complement, not contradict, individual-centered accounts (Isaacs 2014, p. 54; Hormio 2017, p. 20). Assigning moral responsibility to hospitals or other organizations does not mean that their individual members could not be held morally responsible for their actions (List and Pettit 2011, p. 163), and in some contexts choosing a perspective focusing on individuals is justified. For example, if a clinician intentionally neglects hand hygiene guidelines even though they know that this action puts patients at risk, they are individually responsible for this action, independently of the responsibility of the hospital to establish policies for HAI prevention. Vice versa, assigning moral responsibility to a collective does not imply that individuals who are members of the collective are morally responsible for the actions carried out by the collective agent (Isaacs 2014, p. 54). Moreover, even within a structure, groups differ with corresponding tasks and levels of responsibility and individuals can have different obligations. As Isaacs (2011, pp. 149–150) writes:

…once it is clear what is required of the collective, the collective obligation has a derivative impact on the obligations of individuals. Members are, in virtue of their position in a collective with an obligation to act, required to sort out appropriate roles and tasks such that the collective action can take place. The presence of even a putative collective obligation, therefore, begins to impose some order on the actions and obligations of individuals acting in the service of a collective end. To the extent that a means for achieving the end is in reach, the putative collective obligation grounds actual obligations for individuals.

If it is the case that promoting the health of patients is a purpose of a hospital as an organization, individuals working in hospitals have an obligation to act in a way that helps to promote patient health, including the prevention of HAIs. What tasks and responsibilities each individual has, depends on their role in the organization structure (Tuomela and Mäkelä 2020, p. 362). Unlike a hospital cleaner, a medical doctor is not responsible for disinfecting door handles.

Applying the concept of collective responsibility enables ethical evaluation of the dimensions of infection control practices in a way that the individual-centered accounts previously suggested in the literature are not able to do. In particular, it helps to understand how systemic aspects of healthcare, such as employment conditions in hospitals, can count as ethically relevant. If a patient acquires an infection because outsourced cleaning staff have not had enough time to adequately disinfect near-patient surfaces (cf. Dancer and Kramer 2019, p. 3), it is unreasonable to assign moral responsibility to individual cleaners in the case that their precarious employment conditions have impeded them from informing the hospital management of lack of time. In cases like this, not assigning responsibility to the organization that has made the decision to outsource cleaning in order to reduce costs,^14^ i.e., the hospital, would “lead to a deficit of responsibility” (List and Pettit 2011, p. 165).

We suggest that considering hospitals as agents to which collective responsibility can be assigned is useful in the context of discussing the obligation to prevent HAIs. In particular, it enables addressing moral responsibility for systemic factors that the individual-centered accounts discussed in the previous subsection cannot accommodate. Relatedly, it enables making prescriptive statements about hospitals’ obligations to prevent HAIs.

## Conclusion

In this paper, we have argued that the concept of collective responsibility is needed to supplement the existing individual-centered accounts of moral responsibility for HAI prevention. Suggesting that theories of collective responsibility can be fruitful for analyzing cases in medical context is not new (see, e.g., French 1982; Brown and Savulescu 2019; Müller et al. 2021). However, as we have shown, the problem of assigning responsibility for HAI prevention has mostly been approached in individual-centered terms, which has led to a gap in the literature addressing the ethical aspects of infection control. As methods in microbiology have developed and researchers have addressed the systemic determinants of infections, there is more evidence showing that maintaining good hand hygiene is insufficient for preventing the spread of pathogens. Yet the existing individual-centered accounts do not cover factors such as increasing efficiency by streamlining work processes, outsourcing, and understaffing. But it is precisely these factors that determine employment conditions and influence healthcare workers’ ability to act in a way necessary for preventing the spread of infections.

Our analysis suggests that studying the organization of interrelational actions as well as the practices that are closely linked to the working conditions of non-clinical staff are a necessary prerequisite for understanding conditions for patient safety. For example, cleaners are often perceived as support staff and less essential to the core functions of hospitals. Consequently, their work is usually not discussed or reflected in medical ethics or philosophy of medicine. However, as the evidence cited in this paper shows, it has a central role in HAI prevention and merits further study.

To conclude this paper, we will briefly provide a few suggestions for what individual and collective moral responsibility for infection control amount to.^15^ We do not aim for providing detailed recommendations. This is because we believe that concrete guidelines need to be designed to fit the particular characteristics of each healthcare system. Our goal is to create an argumentative support that makes it possible to identify systemic factors that are causally and ethically relevant in infection control.

At the individual-level, adherence to the guidelines applies to all members of a clinical and operational team—without exception (Bosek and Shaner-McRae 2010; Blomberg et al. 2018). The principle of equity implies that the medical doctors, who according to studies comply with the guidelines at a lower rate than other professional groups (e.g., Erasmus et al. 2010, p. 285), must adhere to the rules in the same way as nurses, cleaners, and other employees of the hospitals. The success of infection prevention measures depends on the adherence of all interacting individuals, i.e., is a shared responsibility. It is enough for one actor (at whatever level of responsibility) to disregard the hygiene rule to start the spread of an infection. Individuals should also be willing to report misconduct they observe. In this way, problematic practices can be corrected. Moreover, any obstacles (e.g., missing equipment or cleaning supplies) to carrying out duties necessary for maintaining required hygiene standards should be brought to the attention of those in the position to fix the issue.

At the collective-level, hospitals have an obligation to maintain and promote policies, practices, and structures that enable effective infection prevention measures. For instance, they must provide their employees with adequate resources (e.g., time, equipment) so that these individuals can reasonably be expected to uphold their responsibilities. Hospitals also have an obligation to provide their employers with the adequate training and education opportunities needed to act in a way that science-based guidelines recommend. Moreover, establishing systems enabling anonymous reporting of hygiene misconduct and other issues—in the style of Critical Incident Reporting System already used in healthcare and aviation (Ahluwalia and Marriott 2005)—could promote adherence to rules. Above all, hospitals have an obligation to establish employment and HR practices that do not prevent individuals from acting in a way necessary for stopping the spread of infections. Given what is now known about the systemic determinants of HAIs, a strategic decision that leads to outsourcing or understaffing can be seen to be ethically unsustainable.

Finally, we want to address the worry that the notion of collective responsibility will obscure individuals’ responsibility for preventing HAIs. As we have emphasized above, applying the notion of collective responsibility does not mean that individuals do not have a responsibility for infection control. Instead, it provides a complementary perspective for assessing ethical aspects of infection control. Healthcare workers are responsible for preventing infections, but healthcare organizations also are responsible for providing conditions in which these individuals can act in a way that is required for infection control. Considering collective responsibility thus helps to fill the lacunae left unexplored by individual-centered analyses of moral responsibility.

## Footnotes

1 For critical accounts of the hierarchy of evidence in evidence-based medicine, see, e.g., Stegenga (2014).

2 The review included all empirical studies (n = 96, 65 of which were conducted in intensive care units) assessing compliance rates of healthcare workers who have contact with patients working in general hospital setting that were published in English before January 1st 2009. In the reviewed studies, compliance was measured either by observation or self-report.

3 For a philosophical account of the use of case studies and case reports in research, see Ankeny (2014).

4 Cohorting refers to practice of limiting the number of contacts by assigning each staff member a particular (group of) patient(s) (Nijssen et al. 2003, p. 2785).

5 For example, according to Williams (1993, pp. 55–56), being responsible requires that an agent causes the state of affairs, intends that state of affairs to take place, is in a normal state of mind, and is in a position to response to the state of affairs, i.e. make up for it. How these elements are emphasized depends on the context. List and Pettit (2011, ch. 7), in turn, give three conditions that according to them need to be satisfied for an agent to be held responsible. First of all, the action or a choice in question must have normative significance, “the possibility of doing something good or bad, right or wrong (List and Pettit 2011, p. 155). Second, the agent must have an access to and understanding of evidence that is relevant for making a normative judgment in the case. Third, the agent must be free to choose between different ways of acting. See also Hindriks (2019).

6 It is important to note that if the ignorance concerning consequences of an act is due to the agent’s own culpable action, they can be held responsible for the act. See, e.g., Talbert (2019, Sect. 3.3.4.) for an overview of the discussion on the epistemic condition of moral responsibility.

7 Another contribution to the ethics of hospital infection control is Bryan et al. (2007), who have reviewed ethical frameworks and basic concept with the purpose of providing vocabulary and tools to help infection control professionals make ethical decisions. They do not explicitly address the issue of responsibility.

8 We acknowledge that these principles originating from Beauchamp and Childress’ *Principles of Biomedical Ethics* have been widely criticized, for example for failing to recognize the multiplicity of traditions that exist both within so-called Western cultures and globally (for a brief overview of different lines of criticism, see Huxtable 2013). Unfortunately, we cannot discuss these criticisms here.

9 Millar (2009, p. 236) is an exception as he explicitly asks whether the established causal relationship between how healthcare institutions (e.g., hospitals) function and HAIs implies something with respect to the moral responsibility of hospitals. However, he only poses the question and do not discuss it further.

10 For example, it is possible to assign collective moral responsibility for an act X to a hospital, and at the same time assign moral responsibility for an act Y to a team of medical professionals employed by the hospital.

11 See Lanphier 2021) for a discussion of shared versus collective responsibility for medical information dissemination during a pandemic.

12 It has to be emphasized that according to these authors, not all groups can be morally responsible independently of the responsibilities born by their individual members. In particular, groups that do not have specified structures and decision procedures, such as people who like to attend a particular beach, do not constitute a collective to which collective moral responsibility could be assigned. French (1982, p. 70) calls such groups ‘aggregates’ in contrast to ‘conglomerates’, which are groups that have a decision-making structure and whose identity cannot be reduced to the identities of their members.

13 The account of collective responsibility has been applied in the context of healthcare also by Müller and colleagues (2021) who argued that patient advocacy groups should be understood as collectives capable of intentionality and acting and, thus, count as morally responsible. They have an obligation to advocate for the interests of people suffering from certain diseases, promote research, and encouraging study participation (Müller et al. 2021, p. 8).

14 Organisations’ decisions to contract out certain functions are typically strategic choices motivated by the aim of reducing costs (Litwin et al. 2016, p. 613).

15 The normative standards relevant for making these prescriptions include, for example, ethical principles, existing guidelines, and legal frameworks (Müller et al. 2021, p. 7).

## References

[CR1] Ahluwalia Jag, Marriott Lin (2005). Critical Incident Reporting Systems. Seminars in Fetal & Neonatal Medicine.

[CR2] Ankeny Rachel A (2014). The Overlooked Role of Cases in Causal Attribution in Medicine. Philosophy of Science.

[CR3] Backman Chantal, Zoutman Dick E, Marck Patricia Beryl (2008). An Integrative Review of the Current Evidence on the Relationship between Hand Hygiene Interventions and the Incidence of Health Care-Associated Infections. American Journal of Infection Control, Hand Hygiene and Infection.

[CR4] Beggs Clive B, Noakes Catherine J, Shepherd Simon J, Kerr Kevin G, Andrew Sleigh P, Banfield Katherine (2006). The Influence of Nurse Cohorting on Hand Hygiene Effectiveness. American Journal of Infection Control.

[CR61] Blomberg, Ann-Catrin, Birgitta Bisholt, and Lillemor Lindwall. 2018. Responsibility for patient care in perioperative practice. *Nursing open* 5 (3): 414–421. 10.1002/nop2.153 PMC6056433 30062035

[CR5] Bolton Patrick, McCulloch Timothy J (2018). The Evidence Supporting WHO Recommendations on the Promotion of Hand Hygiene: A Critique. BMC Research Notes.

[CR60] Bosek, Marcia, and Hollie Shaner-McRae. 2010. Hand hygiene as standard practice: Do the rules apply to all healthcare professionals?. *JONA's Healthcare Law, Ethics and Regulation* 12 (4): 101–105. 10.1097/NHL.0b013e3181fcf82b 21116140

[CR6] Brown Rebecca C. H, Savulescu Julian (2019). Responsibility in Healthcare across Time and Agents. Journal of Medical Ethics.

[CR100] Bryan Charles S, Call Theresa J, Elliott Kevin C (2007). The Ethics of Infection Control: Philosophical Frameworks. Infection Control & Hospital Epidemiology.

[CR7] Cassini Alessandro, Plachouras Diamantis, Eckmanns Tim, Sin Muna Abu, Blank Hans-Peter, Ducomble Tanja, Haller Sebastian (2016). Burden of Six Healthcare-Associated Infections on European Population Health: Estimating Incidence-Based Disability-Adjusted Life Years through a Population Prevalence-Based Modelling Study. PLOS Medicine.

[CR8] Clements Archie, Halton Kate, Graves Nicholas, Pettitt Anthony, Morton Anthony, Looke David, Whitby Michael (2008). Overcrowding and Understaffing in Modern Health-Care Systems: Key Determinants in Meticillin-Resistant Staphylococcus Aureus Transmission. The Lancet Infectious Diseases.

[CR9] Collins Stephanie (2013). Collectives’ Duties and Collectivization Duties. Australasian Journal of Philosophy.

[CR62] Dalton, Kathryn R., Clare Rock, Karen C. Carroll, and Meghan F. Davis. 2020. One Health in hospitals: How understanding the dynamics of people, animals, and the hospital built-environment can be used to better inform interventions for antimicrobial-resistant gram-positive infections. *Antimicrobial Resistance & Infection Control* 9 (1): 1–17. 10.1186/s13756-020-00737-2 PMC7268532 32487220

[CR10] Damilare Ogundeji Kolawole (2020). Hand Washing: An Essential Infection Control Practice. International Journal of Caring Sciences.

[CR11] Dancer Stephanie (1999). Mopping up Hospital Infection. Journal of Hospital Infection.

[CR12] Dancer Stephanie (2008). Importance of the Environment in Meticillin-Resistant Staphylococcus Aureus Acquisition: The Case for Hospital Cleaning. The Lancet Infectious Diseases.

[CR13] Dancer Stephanie (2014). Controlling Hospital-Acquired Infection: Focus on the Role of the Environment and New Technologies for Decontamination. Clinical Microbiology Reviews.

[CR14] Dancer Stephanie (2016). Infection Control: Evidence-Based Common Sense. Infection, Disease & Health.

[CR15] Dancer SJ, Kramer A (2019). Four Steps to Clean Hospitals: LOOK, PLAN, CLEAN and DRY. Journal of Hospital Infection.

[CR16] Dancer Stephanie, Adams CE, Smith J, Pichon B, Kearns A, Morrison D (2019). Tracking Staphylococcus Aureus in the Intensive Care Unit Using Whole-Genome Sequencing. Journal of Hospital Infection.

[CR17] Donskey Curtis J (2013). Does Improving Surface Cleaning and Disinfection Reduce Health Care-Associated Infections?. American Journal of Infection Control, Disinfection, Sterilization and Antisepsis: Current Issues, New Research and New Technologies.

[CR18] Elkomy Shimaa, Cookson Graham, Jones Simon (2019). Cheap and Dirty: The Effect of Contracting Out Cleaning on Efficiency and Effectiveness. Public Administration Review.

[CR19] Erasmus Vicki, Daha Thea J, Brug Hans, Richardus Jan Hendrik, Behrendt Myra D, Vos Margreet C, van Beeck EdF (2010). Systematic Review of Studies on Compliance with Hand Hygiene Guidelines in Hospital Care. Infection Control & Hospital Epidemiology.

[CR20] Fee Elizabeth, Garofalo Mary E (2010). Florence Nightingale and the Crimean War. American Journal of Public Health.

[CR21] French Peter A (1982). Collective Responsibility and the Practice of Medicine. The Journal of Medicine and Philosophy: A Forum for Bioethics and Philosophy of Medicine.

[CR22] GadebuschBondio Mariacarla, Kramer A, Assadian O, Exner M, Hübner NO, Scheithauer S, Simon A (2022). Ethische Verantwortung für die Prävention nosokomialer Infektionen. Krankenhaus- und Praxishygiene Hygienemanagement und Infektionsprävention in medizinischen und sozialen Einrichtungen.

[CR23] Gaube Susanne, Tsivrikos Dimitrios, Dollinger Daniel, Lermer Eva (2018). How a Smiley Protects Health: A Pilot Intervention to Improve Hand Hygiene in Hospitals by Activating Injunctive Norms through Emoticons. PLoS ONE.

[CR24] Gilbert Heather A (2020). Florence Nightingale’s Environmental Theory and Its Influence on Contemporary Infection Control. Collegian, Special Issue on Nursing and Health Care History.

[CR25] Gilbert Gwendolyn L, Kerridge Ian (2019). The Politics and Ethics of Hospital Infection Prevention and Control: A Qualitative Case Study of Senior Clinicians’ Perceptions of Professional and Cultural Factors That Influence Doctors’ Attitudes and Practices in a Large Australian Hospital. BMC Health Services Research.

[CR59] Gilbert, Gwendolyn L., Paul Y. Cheung, and Ian B. Kerridge. 2009. Infection control, ethics and accountability. *Medical Journal of Australia* 190 (12): 696–698. 10.5694/j.1326-5377.2009.tb02641.x 19527207

[CR26] Hall L, Mitchell BG, Walker Jimmy (2020). 11—Cleaning and Decontamination of the Healthcare Environment. Decontamination in Hospitals and Healthcare. Woodhead Publishing Series in Biomaterials.

[CR27] Harbarth Stephan, Sudre Philippe, Dharan Sasi, Cadenas Mercedes, Pittet Didier (1999). Outbreak of Enterobacter Cloacae Related to Understaffing, Overcrowding, and Poor Hygiene Practices. Infection Control & Hospital Epidemiology.

[CR28] Herwaldt Loreen A (1996). Ethical Aspects of Infection Control. Infection Control & Hospital Epidemiology.

[CR29] Hindriks Frank (2019). The Duty to Join Forces: When Individuals Lack Control. The Monist.

[CR30] Hormio, Säde. 2017. Marginal Participation, Complicity, and Agnotology: What Climate Change Can Teach us About Individual and Collective Responsibility. PhD Dis., University of Helsinki.

[CR31] Huxtable Richard (2013). For and Against the Four Principles of Biomedical Ethics. Clinical Ethics.

[CR32] Isaacs Tracy (2011). Moral Responsibility in Collective Contexts.

[CR33] Isaacs Tracy (2014). Collective Responsibility and Collective Obligation. Midwest Studies in Philosophy.

[CR34] Jumaa PA (2005). Hand Hygiene: Simple and Complex. International Journal of Infectious Diseases.

[CR35] Lanphier Elizabeth (2021). Physician Outreach During a Pandemic: Shared or Collective Responsibility?. Journal of Medical Ethics.

[CR36] List Christian, Pettit Philip (2011). Group Agency: The Possibility, Design, and Status of Corporate Agents.

[CR37] Litwin Adam Seth, Avgar Ariel C, Becker Edmund R (2017). Superbugs versus Outsourced Cleaners: Employment Arrangements and the Spread of Health Care-Associated Infections. ILR Review.

[CR38] Mathur Purva (2011). Hand Hygiene: Back to the Basics of Infection Control. The Indian Journal of Medical Research.

[CR39] Millar M (2009). Do We Need an Ethical Framework for Hospital Infection Control?. Journal of Hospital Infection.

[CR40] Miller Seumas, Bazargan-Forward Saba, Tollefsen Deborah (2020). Collective Moral Responsibility as Joint Moral Responsibility. The Routledge Handbook of Collective Responsibility.

[CR41] Miracle Vickie A (2008). The Life and Impact of Florence Nightingale. Dimensions of Critical Care Nursing.

[CR42] Mitchell BG, Dancer SJ, Anderson M, Dehn E (2015). Risk of Organism Acquisition from Prior Room Occupants: A Systematic Review and Meta-Analysis. Journal of Hospital Infection.

[CR43] Mortell Manfred (2012). Hand Hygiene Compliance: Is There a Theory-Practice-Ethics Gap?. British Journal of Nursing.

[CR44] Müller Regina, Rach Christoph, Salloch Sabine (2021). Collective Forward-Looking Responsibility of Patient Advocacy Organizations: Conceptual and Ethical Analysis. BMC Medical Ethics.

[CR45] Nijssen Saskia, Bonten Marc J. M, Franklin Cory, Verhoef Jan, Hoepelman Andy I. M, Weinstein Robert A (2003). Relative Risk of Physicians and Nurses to Transmit Pathogens in a Medical Intensive Care Unit. Archives of Internal Medicine.

[CR46] Siani H, Maillard J-Y (2015). Best Practice in Healthcare Environment Decontamination. European Journal of Clinical Microbiology & Infectious Diseases.

[CR47] Silva Mary, Ludwick Ruth (2006). Ethics Column: What Would You Do? Ethics and Infection Control. Online Journal of Issues in Nursing.

[CR48] Smiley, Marion. 2017. “Collective Responsibility”, The Stanford Encyclopedia of Philosophy (Summer 2017 Edition), ed. Edward N. Zalta https://plato.stanford.edu/archives/sum2017/entries/collective-responsibility/

[CR49] Stegenga Jacob (2014). Down with the Hierarchies. Topoi.

[CR50] Talbert, Matthew. 2019. “Moral Responsibility”, *The Stanford Encyclopedia of Philosophy* (Winter 2019 Edition), ed. Edward N. Zalta, https://plato.stanford.edu/archives/win2019/entries/moral-responsibility/

[CR51] Tuomela Raimo, Mäkelä Pekka, Tuomela Raimo, Hakli Raul, Mäkelä Pekka (2020). Group Agents and Their Responsibility. Social Ontology in the Making.

[CR52] Weber David J, Rutala William A, Miller Melissa B, Huslage Kirk, Sickbert-Bennett Emily (2010). Role of Hospital Surfaces in the Transmission of Emerging Health Care-Associated Pathogens: Norovirus, Clostridium Difficile, and Acinetobacter Species. American Journal of Infection Control.

[CR53] Wichsová, Jana, and Andrea Horáková. 2018. Perioperative Ethics and Patient Safety. 10.18662/po/51.

[CR54] Williams Bernard (1993). Shame and Necessity.

[CR55] World Health Organization. 2017a. Evidence of Hand Hygiene as the Building Block for Infection Prevention and Control: An Extract from the Systematic Literature Reviews Undertaken as the Background for the WHO Guidelines on Core Components of Infection Prevention and Control Programmes at the National and Acute Health Care Facility Level (No. WHO/HIS/SDS/2017a.7). World Health Organization.

[CR56] World Health Organization. 2017b. Evidence of Hand Hygiene to Reduce Transmission and Infections by Multidrug Resistant Organisms in Health-Care Settings. https://www.who.int/gpsc/5may/MDRO_literature-review.pdf

[CR57] Zimlichman Eyal, Henderson Daniel, Tamir Orly, Franz Calvin, Song Peter, Yamin Cyrus K, Keohane Carol, Denham Charles R, Bates David W (2013). Health Care-Associated Infections: A Meta-Analysis of Costs and Financial Impact on the US Health Care System. JAMA Internal Medicine.

[CR58] Zuberi Dan (2013). Cleaning Up: How Hospital Outsourcing is Hurting Workers and Endangering Patients.

